# Ocular changes in primary hypothyroidism

**DOI:** 10.1186/1756-0500-2-266

**Published:** 2009-12-29

**Authors:** Banu T Ozturk, Hurkan Kerimoglu, Oguz Dikbas, Hamiyet Pekel, Mustafa S Gonen

**Affiliations:** 1 Department of Ophthalmology, Meram Faculty of Medicine, Selcuk University, Konya, Turkey; 2 Department of Endocrinology and Metabolism Diseases, Sakarya Education and Research Hospital of Ministry of Health, Sakarya, Turkey; 3 Department of Endocrinology and Metabolism Diseases, Meram Faculty of Medicine, Selcuk University, Konya, Turkey

## Abstract

**Background:**

To determine the ocular changes related to hypothyrodism in newly diagnosed patients without orbitopathy.

**Findings:**

Thirty-three patients diagnosed to have primary overt hypothyroidism were enrolled in the study. All subjects were assigned to underwent central corneal thickness (CCT), anterior chamber volume, depth and angle measurements with the Scheimpflug camera (Pentacam, Oculus) and cup to disc ratio (C/D), mean retinal thickness and mean retinal nerve fiber layer (RNFL) thickness measurements with optical coherence tomography (OCT) in addition to ophthalmological examination preceeding the replacement therapy and at the 1^st^, 3^rd ^and 6^th ^months of treatment.

The mean age of the patients included in the study were 40.58 ± 1.32 years. The thyroid hormone levels return to normal levels in all patients during the follow-up period, however the mean intraocular pressure (IOP) revealed no significant change. The mean CCT was 538.05 ± 3.85 μ initially and demonstrated no statistically significant change as the anterior chamber volume, depth and angle measurements did. The mean C/D ratio was 0.29 ± 0.03 and the mean retinal thickness was 255.83 ± 19.49 μ initially and the treatment did not give rise to any significant change. The mean RNFL thickness was also stable during the control visits, so no statistically significant change was encountered.

**Conclusions:**

Neither hypothyroidism, nor its replacement therapy gave rise to any change of IOP, CCT, anterior chamber parameters, RNFL, retinal thickness and C/D ratio.

## Introduction

The eye is a unique sensory organ that is prone to the effects of various systemic disorders. Hypothyroidism is one of these, presenting frequently with chemosis, periorbital edema and blepharoptosis. These changes, described as orbitopathy are attributed to the accumulation of hydrophilic mucopolysaccharides in the ground substance of dermis and other tissues leading to thickening which is called myxoedema [1].

Another proposed, ocular finding of hypothyroidism is the intraocular pressure (IOP) rise. There has been a number of reports regarding the higher prevalance of primary open angle glaucoma (POAG) among hypothyroid individuals, however contoversy stil exists as some others have failed to demonstrate this [2-5]. In a study of Smith et al [2] 23.4% of POAG patients had hypothyroidism. As they noted decrease of IOP with treatment of hypothyroidism in a patient with POAG, IOP rise was attributed to the reduction in facility of outflow in the hypothyroid state [6,7]. Another study of Centanni et al [8] demonstrated a reversible increase of IOP even in subclinical hypothyroidism which raised the question about whether some microscopic findings precede the macroscopic signs of hypothyroidism. The study of Bahçeci et al [9] supported this further by demonstrating a reversible increase of the central corneal thickness (CCT) which lead to a reversible IOP rise and a decreased CCT correlated with the decrease of thyroid-stimulating hormone (TSH).

In an attempt to elucidate the precise effect of hypothyroidism on ocular structures including cornea, anterior chamber, lens and retina, we conducted a study on newly diagnosed, overt hypothyroid patients without orbitopathy comparing the pre- and postreplacement findings.

## Materials and methods

This prospective, single-center, clinical study was conducted at the department of Ophthalmology, Selcuk University, Meram Faculty of Medicine with the collaboration of the department of Endocrinology. Sixtysix eyes of 33 patients who were diagnosed to have acquired primary hypothyroidism in the outpatient clinic of the Endocrinology department were enrolled in the study. The initial ophthalmological examination was conducted preceding the reatment of hypothyroidism to exclude any history or finding of an ocular disease and for the approval of the informed consent. A complete ophthalmic examination including visual acuity, IOP with Goldmann applanation tonometer, anterior segment and fundus examination was performed and patients with any sign of orbitopathy, corneal pathology, glaucoma and retinal vascular disease were excluded from the study. Eligible patients were assigned to undergo CCT, anterior chamber volume, depth and angle with the Scheimpflug camera (Pentacam, Oculus) in addition to cup to disc ratio (C/D), retinal thickness and retinal nerve fiber layer (RNFL) thickness measurements with optical coherence tomography [(OCT), (Stratus OCT-3) Carl Zeiss Meditec, Inc., CA]. After pupillary dilatation, fast optic disc and fast RNFL scans were performed by OCT. C/D ratios were recorded after optic disc analysis was carried out. The retina thickness in the superior, nasal, inferior and temporal quadrants, calculated automatically by OCT device were recorded and average RNFL thickness values are obtained from the retinal nerve fiber analysis of OCT.

Control visits were scheduled for the first, 3rd and 6th months after initiation of the medical therapy for hypothyrodism and included the same ophthalmic examination procedures performed initially together with Pentacam and OCT measurements following control examination in the outpatient clinic of the Endocrinology department and thyroid hormone level measurements including thyroid-stimulating hormone (TSH), free T_3 _and free T_4 _in the clinic of endocrinology. To avoid the effect of diurnal and personel changes, both the inital examination and control visits were performed at the same time in the morning and by the same doctor (BTO).

The data was analyzed by using the Statistical Package for Social Science (SPSS) programme (Worldwide Headquarters SPSS Inc. 15.0 Windows package program). According to the normality tests, parameters showing a normal distribution were analyzed using the repeated measures test and the remaining parameters showing an abnormal distribution were analyzed using the nonparametric k related sample test. Additionally the correlation of the TSH change with all of the study parameters were analyzed by Pearson correlation test and Spearman's correlation test depending on the distribution type of the data. As TSH has been demonstrated to be an excellent screening test for hypothyroidism correlation with thyroid hormones has not been analyzed [1]. A p value of < 0.05 was considered as statistically significant.

## Results

Sixty-six eyes of 32 female patients and 1 male patient who was diagnosed to have primary clinical hypothyroidism and completed the sixth month follow-up program were enrolled in the study. The mean age was 40.58 ± 1.32 years ranging between 19-61 years. The initial mean TSH level of 16.02 ± 2.38 μIU/ml decreased to 3.36 ± 0.54 μIU/ml at the end of the sixth months, while mean free T_3 _level increased from 2.21 ± 0.19 pg/ml to 4.05 ± 0.07 pg/ml and mean free T_4 _level achieved a normal level of 1.06 ± 0.06 ng/dl by ascending from the initial level of 0.57 ± 0.02 ng/dl (Table 1). Hypothyroidism was related to Hashimoto thyroiditis in 15 out of 33 patients.

**Table 1:** Pre- and posttreatment thyroid hormone levels

	Pretreatment	1st Month	3rd Month	6th Month	Normal range
TSH (μIU/ml)	16.02 ± 2.38	6.63 ± 0.83	4.14 ± 0.70	3.36 ± 0.54	0.4-4.0

Free T_3 _(pg/ml)	2.21 ± 0.19	3.45 ± 0.14	3.85 ± 0.19	4.05 ± 0.07	1.57-4.71

Free T_4 _(ng/dl)	0.57 ± 0.02	0.75 ± 0.03	1.02 ± 0.07	1.06 ± 0.06	0.8-1.90

The IOP measured at each control visit was within normal limits in all patients except one patient. She was diagnosed to have ocular hypertension and warranted use of both timolol acetate and dorzolamide hydrochloride. This patient was discarded during the mean IOP calculations. The mean IOP was 14.40 ± 2.45 mmHg initially, 14.42 ± 2.76 at the first month, 14.22 ± 2.97 mHg at the third month and 14.69 ± 2.17 mmHg at the 6th month control. These measurements were also analysed statistically, but any significant IOP decrease with treatment was lacking (p = 0.23) and IOP measurements was not correlated with the decrease in TSH (p = 0,10).

The mean CCT obtained via Pentacam Scheimpflug camera was 538.05 ± 31.29 μ before the treatment. It demonstrated a slight decrease at the end of the first month of therapy, followed by a slight increase at the 3^rd ^month control and remained quite stabile at the 6^th ^month control at a level of 537.64 ± 33,37 μ. Statistical analysis revealed no significant change of the CCT (p = 0.82). The mean anterior chamber depth changed from the initial level of 2.90 ± 0.38 mm to 2.89 ± 0.38 mm at the end of the first month, 2.89 ± 0.38 mm at the third month control and 2.87 ± 0.30 mm at the sixth month control. This gradual decrease was also found to be statistically insignificant (p = 0.31). In contrast, both the mean volume of anterior chamber which was 159.67 ± 43.91 mm^3 ^at the beginning and the mean anterior chamber angle measured as 35.76 ± 7.58° at the initial visit demonstrated variation. However the statistical analysis revealed no significant change for both parameters (p = 0,53, p = 0.86, respectively) (Table 2).

**Table 2:** Change of the follow-up parameters measured with Pentacam Scheimpflug camera at control visits

	Pretreatment	1st Month	3rd Month	6th Month	p
Central corneal thickness (μ)	538.04 ± 31.29	536.95 ± 32.33	537.04 ± 32.86	537.64 ± 33.37	0.82

Anterior chamber depth (mm)	2.90 ± 0.38	2.89 ± 0.38	2.89 ± 0.38	2.87 ± 0.40	0.31

Anterior chamber volume (mm^3^)	159.67 ± 43.91	155.32 ± 45.43	159.38 ± 41.88	158.62 ± 44.28	0.53

Anterior chamber angle (°)	35.76 ± 7.58	35.15 ± 7.01	35.20 ± 7.63	35.41 ± 7.61	0.86

The correlation between the change of the TSH level and the change of the CCT, anterior chamber depth, volume and angle was evaluated by comparing the initial and the final (6^th ^month) measurements however no significant correlation could be estimated (p = 0.43, p = 0.31, p = 0.40, p = 0.49 respectively).

The mean retinal thickness, mean RNFL thickness and the mean C/D ratio was 255.83 ± 19.49 μ, 97.71 ± 1.83 μ and 0.29 ± 0.03 respectively at the beginning of the study and no significant change could be detected after treatment according to the statistical analysis (p = 0.79, p = 0.68, p = 0.43, respectively) (Table 3). The correlation of retinal thickness, RNFL thickness and C/D ratio measurement with the decrease of TSH was also insignificant (p = 0.14, p = 0.13, p = 0.39)

**Table 3:** Change of the follow-up parameters measured with OCT at control visits

	Pretreatment	1st Month	3rd Month	6th Month	p
Mean Nerve Fiber Layer Thickness (μ)	97.71 ± 14.86	98.70 ± 12.05	98.97 ± 10.59	99.29 ± 10.32	0.79

Cup/Disc Ratio	0.29 ± 0.27	0.30 ± 0.27	0.30 ± 0.26	0.30 ± 0.27	0.68

Retinal Thickness	255.83 ± 19.49	258.63 ± 20.38	258.81 ± 17.33	259.05 ± 18.03	0.43

## Discussion

Thyroid hormone plays a pivotal role in the neural development of the eye especially for normal development of retina and attainment of color vision. It regulates intrinsic mechanisms for controlling retinal cytoarchitecture and layering [10]. As Gamborino et al. [11] demonstrated in a rat model, the photoreceptor and ganglion cell layer thickness displayed significantly lower values in congenital-neonatal hypothyroidism. In contrast acquired deficiency of thyroid hormones is reported to mainly affect the IOP beside findings of orbitopathy like periorbital edema and chemosis related to myxedema [1].

The first reports regarding the IOP increase in hypothyroidism are dating back to 1897 and has been ascribed to hypothalamic disturbance either directly or via the pituitary gland acting on the thyroid and the eye at the same time and glaucoma has been associated at times with thyrotoxicosis and at times with myxoedema. Another speculation of Cheng & Perkins is the genetic predisposition to both conditions [12]. In 1965, McLenachan and Davies postulated that the deposition of glycosaminoglycan in trabecular meshwork might lead to a decrease of aqueus humor outflow [13], however it has not been demonstrated so far. Later Becker et al [14] raised the question whether hypothyroidism induce myxedema of the trabecular meshwork and Smith et al [15] proposed vasculopathy altering ocular bloodflow as the mechanism of IOP increase in hypothyroidism. The glycosaminoglycan deposition in trabecular meshwork seems to gain most attention among these, though it could not been demonstrated histopathologically so far.

The literature contains a number of conflicting reports [2-4,15] that determine the incidence of hypothyroidism among POAG patients to decide whether screening of thyroid hormones are necessary for open-angle glaucoma patients or not. Smith et al [2] and Girkin et al [4] found a high incidence of hypothyroidism among subjects with POAG, whereas Gillow [15] and Munoz-Negrete [3] failed to show any evidence of a clinically important association between hypothyroidism and glaucoma as Cheng and Perkins [13].

Other type of studies designed to estimate the prevalance of open-angle glaucoma in acquired hypothyroidism patients found no relationship between these disorders, except Tahat et al [16] who reported a positive relationship, though only 3 of 60 patients was diagnosed to have glaucoma. Karadimas et al [5] examined 100 hypothyroid patients and none of them had glaucoma. In our study, we found ocular hypertension in only one patient. As it is to our knowledge glaucoma incidence increases over 40 years [17]. Studies evaluating the incidence of hypothyrodism among glaucoma patients have usually older age ranges, in contrast studies designed to determine the incidence of glaucoma among hypothyroid patients have younger subjects which may explain the lower glaucoma incidence found in this type of studies including ours.

As estimating only the prevalance of glaucoma may cause to overlook the true effect of hypothyroidism on IOP because changes under 21 mmHg would not be taken into account; we investigated the correlation of IOP with the change of TSH and found no decrease of IOP parallel to the decrease of TSH with treatment. Bahçeci et al [9] found significant decrease of IOP with treatment, however it was also not correlated with the changes in the thyroid hormone levels. They also reported a significant decrease of CCT after replacement therapy and stated that these reversible changes may be related to mucopolysaccharide deposition in corneal stroma as corneal thickness decreased following the replacement therapy, however the mean CCT revealed no significant change in our study. Both studies included newly detected hypothyroid patients however the period of hypothyroidism is unknown and the method for corneal thickness measurements are different. Bahceci et al used ultrasonic pachymetry while we used the Scheimpflug camera. These may be an explanation of the conflicting results.

In our study we aimed to sort out the effect of hypothyroidism on ocular tissues including cornea, anterior chamber, lens and retina in search for an answer to these contradictory results and evaluated the pre- and posttreatment measurements of the anterior chamber depth, volume, angle of hypothyroid patients. The initial measurements were not significantly different from the ones at the 1st, 3rd and 6th months of replacement therapy. This finding may be explained by the lack of orbitopathy and unknown duration of the disease. However as our study is the first evaluating these parameters, further studies comparing these findings with that of the hypothyroid patients with orbitopathy are necessary.

The changes in RNFL thickness, C/D ratio and retinal thickness were also evaluated in this study and found steady in all measurements during the follow-up period. In the literature RNFL thickness of hypothyroid patients was obtained just in the study of Bahçeci et al [9] and they also found no change in RNFL thickness between pretreatment and postreplacement measurements.

## Conclusions

Regarding these findings together, this survey suggest that a direct influence of thyroid hormones on ocular structures is unlikely. Though the unknown period of the hypothyroid state, the younger mean age and lack of orbitopathy might be responsible for the conflicting results, it may also support the speculation about the predisposition of individuals with inclination to auto-immunity to several diseases including hypothyroidism and glaucoma [18]. Further studies evaluating hypothyroid patients according to etiology (Hashimoto disease, Graves disease), period of the disease and presence of orbitopathy seperately may enlighten this dilemma.

## Competing interests

The authors declare that they have no competing interests.

## Authors' contributions

BTO carried out the ophthalmological examinations and drafted the manuscript. HK participated in its design and coordination. OD carried out the endocrinological examinations. SG participated in its design and coordination. HP revised the manuscript for publication. All authors read and approved the final manuscript.
